# Decreased NK-cell tumour immunosurveillance consequent to JAK inhibition enhances metastasis in breast cancer models

**DOI:** 10.1038/ncomms12258

**Published:** 2016-07-13

**Authors:** Alessia Bottos, Dagmar Gotthardt, Jason W. Gill, Albana Gattelli, Anna Frei, Alexandar Tzankov, Veronika Sexl, Aleksandra Wodnar-Filipowicz, Nancy E. Hynes

**Affiliations:** 1 Friedrich Miescher Institute for Biomedical Research, Maulbeerstrasse 66, CH-4058 Basel, Switzerland; 2 Institute of Pharmacology and Toxicology, Department for Biomedical Sciences, University of Veterinary Medicine, 1210 Vienna, Austria; 3 University of Basel, CH-4002 Basel, Switzerland; 4 Institute of Pathology, University Hospital Basel, CH-4031 Basel, Switzerland; 5 Stem Cell Center of Competence, University of Basel, CH-4002 Basel, Switzerland

## Abstract

The JAK/STAT pathway is an attractive target for breast cancer therapy due to its frequent activation, and clinical trials evaluating JAK inhibitors (JAKi) in advanced breast cancer are ongoing. Using patient biopsies and preclinical models of breast cancer, we demonstrate that the JAK/STAT pathway is active in metastasis. Unexpectedly, blocking the pathway with JAKi enhances the metastatic burden in experimental and orthotopic models of breast cancer metastasis. We demonstrate that this prometastatic effect is due to the immunosuppressive activity of JAKi with ensuing impairment of NK-cell-mediated anti-tumour immunity. Furthermore, we show that immunostimulation with IL-15 overcomes the enhancing effect of JAKi on metastasis formation. Our findings highlight the importance of evaluating the effect of targeted therapy on the tumour environment. The impact of JAKi on NK cells and the potential value of immunostimulators to overcome the weakened tumour immunosurveillance, are worthwhile considering in the clinical setting of breast cancer.

## Abstract (web summary)

JAK inhibitors are currently undergoing evaluation in clinical trials for advanced breast cancer. Here, the authors show that JAK pathway inhibition increases metastasis in mouse models of breast cancer by impairing NK anti-tumour activity and that these side effects can be overcome by addition of IL-15.

## Graphical abstract

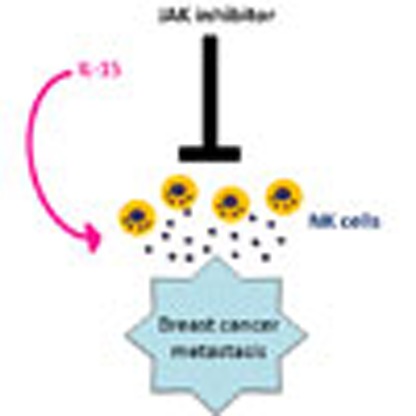

The signal transducer and activator of transcription (STAT) protein family plays a major role in cancer^1^. Aberrant activation of STATs, especially of STAT3, contributes to tumour progression at several levels. STATs regulate the transcription of target genes controlling tumour cell proliferation and differentiation, as well as genes encoding proteins with major roles in conditioning the tumour microenvironment, for instance, by controlling angiogenesis and the recruitment of immune cells^1^,^2^. In breast cancer, STAT3 and STAT5 activation, assessed by phosphorylation on specific tyrosine residues, is frequently observed in the cancer cells; with STAT3 often activated in invasive and metastatic tumours^3^,^4^. Among the plethora of kinase receptors that stimulate STATs, janus kinases (JAK), in particular JAK2 driving STAT3 and STAT5 activation, have been reported to have significant roles in breast cancer. For example, the activation of JAK2/STAT3 signalling by interleukin (IL)-6 regulates the growth and maintenance of stem-like breast cancer cells (CD44^+^CD24^−^; ref. 5). Moreover, active JAK2/STAT5 signalling in triple-negative breast cancer is one mechanism causing resistance to PI3K/mTOR inhibition^6^. With the rationale that sub-types of breast tumours show activation of the JAK/STAT pathway, JAK inhibitors (JAKi) recently developed to treat haematological disorders^7^,^8^,^9^ are currently undergoing evaluation in clinical trials for advanced breast cancer^10^. An important, not yet understood, aspect of this therapeutic approach is its impact on metastasis, which is the major cause of cancer-associated death^11^. In breast cancer, metastatic spread of tumour cells to the bone is frequent and an important cause of mortality^12^.

A major problem in treating metastatic disease is that disseminated tumour cells show fundamental biological and molecular differences compared with the primary tumour^13^. This can be due to acquired resistance to targeted therapy or to environmental features of the metastatic site, where the surrounding stroma can drive the clonal selection of cancer cells, influence the dormancy/proliferation of disseminated tumour cells and hinder therapeutic response^14^,^15^,^16^,^17^. Immune cells add an additional layer of complexity to the crosstalk between cancer cells and the tumour microenvironment^18^,^19^. Evasion from immunosurveillance is one of the hallmarks of cancer^20^ and lymphocytes (T cells, natural killer (NK) cells and NKT cells) have pivotal roles in the recognition and elimination of tumour cells by the immune system^21^. Indeed, clinical studies have shown that the presence of tumour-infiltrating lymphocytes (TIL) within the tumour is associated with better prognosis in breast and other solid cancers^22^,^23^,^24^,^25^.

NK cells are a component of the innate immune response and are responsible for the rapid recognition and elimination of cancer cells^26^. NK-cell cytolytic activity is tightly regulated by a complex system of activating and inhibitory receptors that control the recognition of target cells. A common mechanism for tumour cell clearance by NK cells is the release of cytotoxic granules containing perforin and granzymes, which induce cancer cell death^27^. Several cytokines essential for NK-cell development, maturation, and activation (such as IL-15, IL-12 and IL-21), utilize JAKs to signal through STATs^28^. Importantly, preclinical studies examining the role of the JAK/STAT pathway in NK cells revealed a multifaceted role for STATs in controlling the anti-cancer activity of NK cells. For example, inhibition of STAT3, which has an immunosuppressive effect, enhances NK-cell-mediated cytotoxicity^29^,^30^. On the other hand, STAT1, STAT4 and STAT5 are essential for the development of efficient NK-cell anti-tumour surveillance^31^,^32^,^33^,^34^. With the rationale in mind that the JAK/STAT pathway controls key aspects of the innate tumour immunity, it becomes very important to understand how metastasis formation is influenced by treatment with JAKi. The results we present here show that inhibition of the JAK pathway, despite blocking STAT activation in tumour cells, enhances metastatic burden in preclinical models of breast cancer by decreasing NK-cell-mediated anti-tumour immunity.

## Results

### JAK/STAT is active in breast cancer bone metastasis

**Figure 1:**
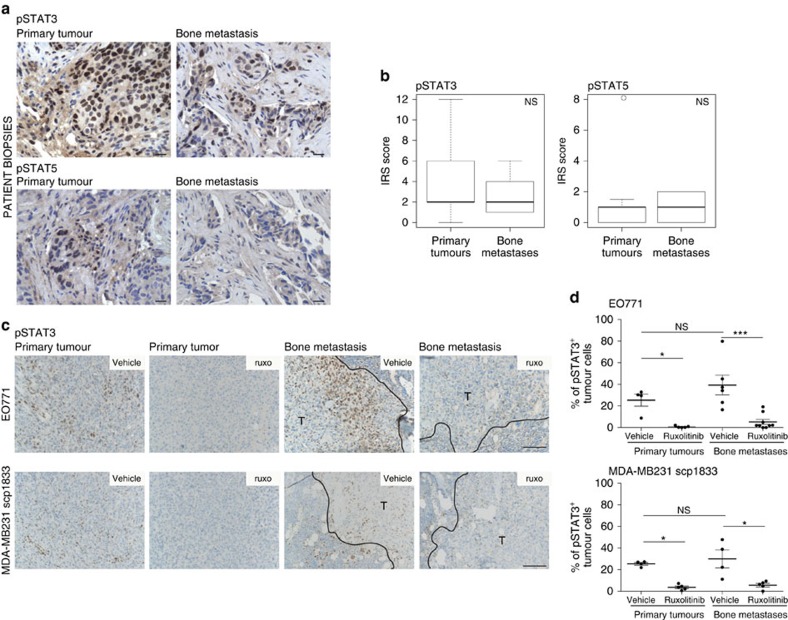
The JAK/STAT pathway is active in breast cancer bone metastasis. (**a**,**b**) pSTAT3 and pSTAT5 analysis by immunohistochemistry (IHC) in primary tumours and paired bone marrow metastases from 14 breast cancer patients. (**a**) Images of pSTAT3 and pSTAT5 (patient number 13) in the primary tumour and the paired bone metastasis are shown. Scale bar, 20 μm. (**b**) Quantification of pSTAT3 and pSTAT5 immunoreactive score (IRS) in primary tumours and paired bone marrow metastases. The boxplots show the median, the 25th and 75th percentiles and the minimum and maximum values after outlier removal. *n*=14 patients. pSTAT3 and pSTAT5 IRS score of primary tumours and bone metastases did not show significant differences (NS) using the exact Wilcoxon's rank-sum test. (**c**,**d**) JAK/STAT pathway activation in EO771 and 1833 breast cancer models. (**c**) Representative images of pSTAT3 staining in EO771 and 1833 primary mammary tumours and bone metastases from mice treated with vehicle or ruxolitinib (90 mg kg^−1^ BID). T, tumour area. Scale bar, 100 μm. (**d**) Quantification of percentage of positive pSTAT3 tumour cells in primary tumours and bone metastases from EO771 and 1833 tumour models from mice treated with vehicle or ruxolitinib (90 mg kg^−1^ BID). The percentage of positive pSTAT3 tumour cells from individual mice and the mean±s.e.m. are shown. For EO771 primary tumours vehicle=4, ruxolitinib=5; bone metastases vehicle=6, ruxolitinib=9 from two independent experiments. For 1833 primary tumours vehicle=4, ruxolitinib=5; bone metastases vehicle=4, ruxolitinib=5. *P<0.05, ***P<0.005, NS, not significant with two-tailed Mann–Whitney test.

The JAK kinase signal transducers STAT3 and STAT5 are frequently active in human breast cancers^3^,^5^, but their activation status in tumours colonizing the bone, remains unknown. We examined the level of tyrosine phosphorylated (p)STAT3 and pSTAT5 in clinical samples from primary breast cancers and paired bone metastases (Supplementary Table 1). 93% and 57% of primary tumours were positive for pSTAT3 and pSTAT5, respectively. In bone metastases, the pattern resembled the primary tumours, with activation of STAT3 prevailing over STAT5. Moreover, similar pSTAT levels were found in primaries and metastases (Fig. 1a,b). These data indicate that STATs might represent a therapeutic target in metastatic disease.

Having established that STATs are active in human bone metastasis, our next goal was to experimentally study the role of the JAK/STAT pathway in breast cancer models. For this, we employed preclinical models of bone metastatic breast tumours, taking advantage of the bone tropism of the human MDA-MB231 scp1833 (1833) (ref. 16) and the mouse EO771 cancer cell lines, when injected via the intracardiac route. In both models STAT3, but not STAT5, was activated *in vitro* (Supplementary Fig. 1A) and *in vivo,* in primary tumours and bone metastasis (Fig. 1c,d; Supplementary Fig. 1B). Treatment with the JAK1/2 inhibitor ruxolitinib^35^ blocked IL6-mediated STAT3 activation *in vitro* (Supplementary Fig. 1A) and decreased pSTAT3 levels in primary tumours and in bone metastases (Fig. 1c,d). These data demonstrate that the STAT pathway is active in breast cancer cells in the bone metastatic site and it is targetable by JAKi.

### Inhibition of JAK/STAT enhances bone metastasis

**Figure 2:**
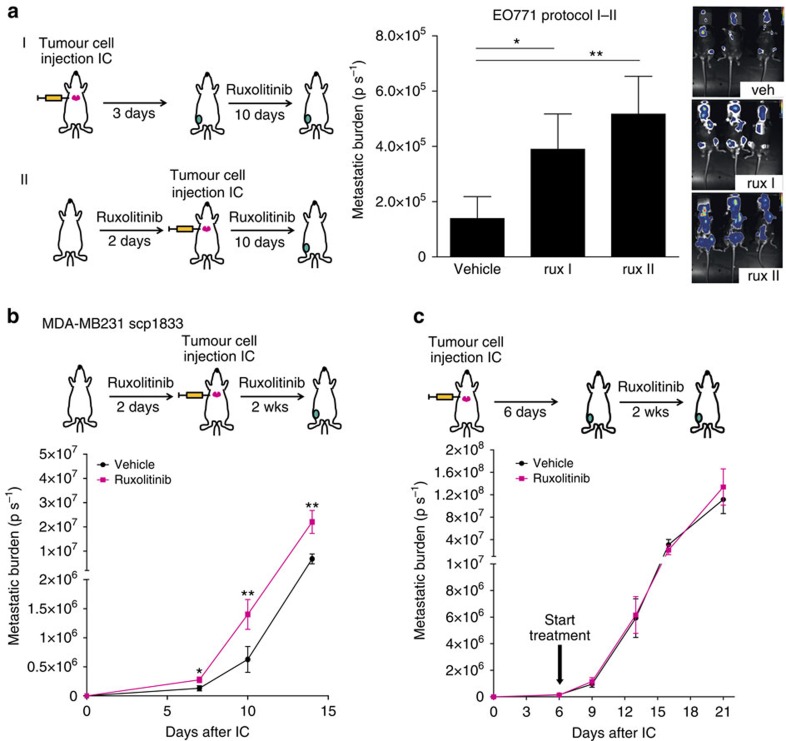
JAKi enhances breast cancer bone metastasis. (**a**) Left: scheme of the protocols I and II. Right:quantification of bone marrow metastases in mice intracardially (IC) injected with 0.5 × 10^6^ EO771 cells and treated with vehicle or ruxolitinib (90 mg kg^−1^ BID). Treatment was started 3 days after tumour cell injection (rux I) or 2 days before tumour cell injection (rux II) and continued for 10 days. Bars show mean±s.e.m. of bioluminescence (total flux (p s^−1^)) quantified in long bones positive for metastases. Representative images of mice treated for 10 days with vehicle or ruxolitinib are shown. Bone number analysed at day 10: vehicle *n*=10, ruxolitinib I *n*=14, ruxolitinib II *n*=15. (**b**) 1.3 × 10^5^ 1833 cells were injected into athymic nude mice pretreated for 2 days with ruxolitinib (90 mg kg^−1^ BID), treatment continued for 14 days and metastases were quantified in the long bones. The graph shows mean±s.e.m. of bioluminescence (total flux (p s^−1^)) in long bones positive for metastasis at different time points. Bone number analysed: vehicle *n*=10–12, ruxolitinib *n*=12–14. (**c**) 1.3 × 10^5^ 1833 cells were injected into athymic nude mice. After 6 days, treatment with vehicle or ruxolitinib (90 mg kg^−1^ BID) started and continued for 2 weeks. The graph shows mean±s.e.m. of bioluminescence (Total Flux (p s^−1^)) in long bones positive for metastasis at different time points. Bone number analysed: vehicle *n*=13–14, ruxolitinib *n*=10-11. *P<0.05, **P<0.01 with two-tailed Mann–Whitney test.

**Figure 3:**
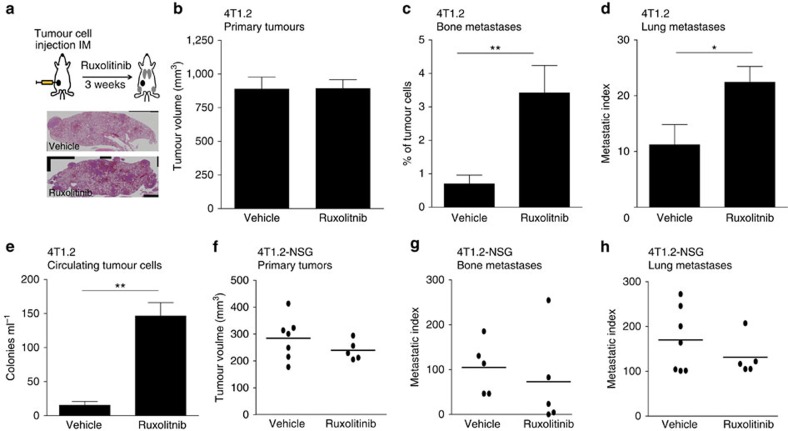
JAKi enhances metastasis dissemination by impairing anti-tumour immunity. (**a**) Scheme of the experimental procedure: 1x10^5^ 4T1.2 tumour cells were injected intramammary (IM), into the mammary fat pad of Balb/c mice and animals were treated for 3 weeks with vehicle or ruxolitinib (90 mg kg^−1^ BID). Representative images of H&E used for lung metastasis quantification are shown. (**b**) Quantification of primary mammary tumour volume (mm^3^). Data are expressed as the mean±s.e.m. Vehicle *n*=10, ruxolitinib *n*=9. (**c**) Quantification of spontaneous bone metastases. Bars represent the mean±s.e.m of percentage of tumour cells (CD45^−^ TR119^−^ EPCAM^+^) in the whole bone quantified by FACS. Vehicle *n*=7, ruxolitinib *n*=6. (**d**) Quantification of spontaneous lung metastases. Bars represent the mean±s.e.m of the metastatic index (area of metastasis/area of lung). Vehicle *n*=7, ruxolitinib *n*=6. (**e**) Quantification of circulating tumour cells. Bars represent mean±s.e.m of the colony number per ml of blood. Vehicle *n*=7, ruxolitinib *n*=5. (**f**–**h**) 1x10^5^ 4T1.2 tumour cells were injected IM in NSG mice and animals were treated for 16 days with ruxolitinib (90 mg kg^−1^ BID). (**f**) Quantification of primary mammary tumour volume (mm^3^). The tumour volume in individual mice and the mean are shown. Vehicle *n*=7, ruxolitinib *n*=5. (**g**) Quantification of spontaneous bone metastases. The bone metastatic index (number of colonies per primary tumour volume) from individual mice and the mean are shown. Vehicle *n*=5, ruxolitinib *n*=5. (**h**) Quantification of spontaneous lung metastases. The metastatic index (number of nodules in the big lobe/primary tumour volume) from individual mice and the mean are shown. Vehicle *n*=7, ruxolitinib *n*=5. *P<0.05, **P<0.01 with two tailed Mann–Whitney test.

We tested the effect of ruxolitinib on bone metastasis in the EO771 model following two protocols (Fig. 2a): starting treatment shortly after cancer cell injection (I) or preconditioning the environment before injecting tumour cells (II). Unexpectedly, JAK inhibition profoundly increased tumour burden in the bone, with the strongest effect in pretreated mice (Fig. 2a rux II). Ruxolitinib caused the same effect in the 1833 model, with a significant difference in tumour burden appearing early (7 days) after tumour cell inoculation (Fig. 2b). However, when treatment was started only after establishment of metastasis (6 days), JAK inhibition did not enhance growth of tumour cells in the bone (Fig. 2c). Notably, in both EO771 and 1833 models, neither primary tumour growth nor *in vitro* proliferation was affected by ruxolitinib (Supplementary Fig. 2A,B). To investigate the effect of JAKi on tumour cell dissemination we employed the 4T1.2 orthotopic model, which shows STAT3 activation (Supplementary Fig. 2C), and metastasizes to the lungs and the bone from the primary tumour (Fig. 3a). 4T1.2 primary tumour growth was not affected by ruxolitinib (Fig. 3b), but there was a significant increase of tumour cells in the bone (Fig. 3c). Moreover, ruxolitinib enhanced lung metastasis (Fig. 3a,d) and the number of circulating tumour cells (Fig. 3e), indicating that JAKi generally increase metastatic spread. Taken together, these data indicate that early steps of metastatic dissemination and colonization, and not tumour cell proliferation, are enhanced by JAK pathway inhibition, and that preconditioning of the host environment plays a role in JAKi-induced metastasis.

### JAKi enhances metastasis by impairing anti-tumour immunity

To understand the mechanism underlying the increase in metastasis, we considered the host immune system as a bystander target of JAKi. Indeed, the JAK/STAT pathway is utilized by numerous cytokines that regulate the development and function of lymphocytes^2^,^36^. To investigate this, we analysed the effect of ruxolitinib on metastatic dissemination in severely immunocompromised NOD-SCID gamma (NSG) hosts, which lack mature T cells, B cells and NK cells. 4T1.2 primary tumour growth was not affected (Fig. 3f) and, remarkably, no increase in bone or lung metastases was observed in ruxolitinib-treated NSG mice (Fig. 3g,h), providing evidence that JAK/STAT pathway inhibition increases metastasis by interfering with anti-tumour immunity.

To investigate the importance of the JAK/STAT pathway in the immune compartment, we analysed the activation of STATs in TILs within the tumour tissue of our cohort of breast cancer patients. pSTAT3 and pSTAT5 were detected in TILs (Supplementary Fig. 3A), suggesting that the JAK pathway is active in the tumour stroma compartment. The TIL score in metastasis was independent from its level in the primary tumour (Supplementary Fig. 3B) and STAT activation in the cancer cells was not predictive for TIL infiltration (Supplementary Fig. 3C). However, we observed a significant reduction of TILs in bone metastasis, compared to the primary tumour (Supplementary Fig. 3B), reinforcing the idea that cancer cells evade the immune system in order to disseminate and grow in distant organs.

### JAKi decrease NK-cell-mediated immunosurveillance

**Figure 4:**
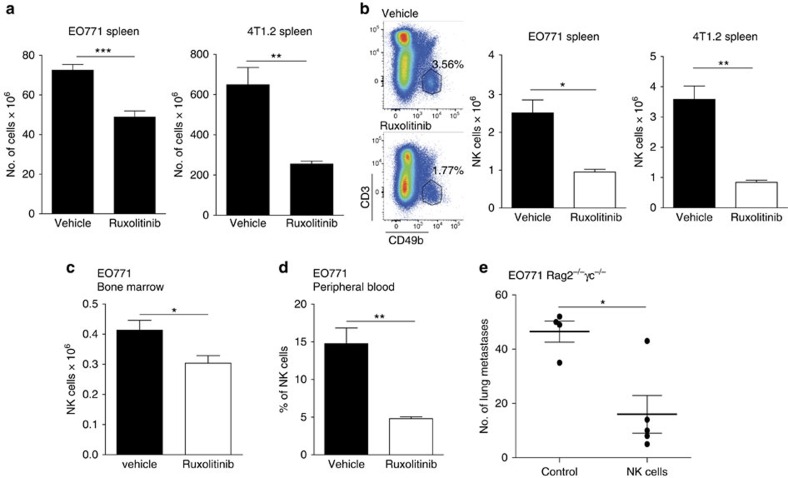
JAKi enhance metastasis by decreasing the NK-cell population. (**a**) Quantification of total spleen cell number in EO771 and 4T1.2 tumour-bearing mice treated with vehicle or ruxolitinib (90 mg kg^−1^ BID). Bars show mean±s.e.m. For EO771: vehicle *n*=7, ruxolitinib *n*=8; for 4T1.2: vehicle=7, ruxolitinib=6. (**b**) Left: representative images of NK-cell-gating for FACS analysis of EO771 spleens. Right: quantification of NK cells (CD3^−^ CD49b^+^) in spleens from EO771 tumour-bearing mice and of NK cells (CD3^−^ CD49b^+^NKp46^+^) in spleens from 4T1.2 tumour-bearing mice, after treatment with vehicle or ruxolitinib (90 mg kg^−1^ BID). Bars show the mean±s.e.m. For EO771: vehicle *n*=7, ruxolitinib *n*=8; for 4T1.2: vehicle=7, ruxolitinib=6. (**c**) Quantification of NK cells (CD3^−^ CD49b^+^) in the bone marrow of EO771 tumour-bearing mice treated with vehicle or ruxolitinib (90 mg kg^−1^ BID). Bars show the mean±s.e.m. Vehicle *n*=7, ruxolitinib *n*=8. (**d**) Quantification of NK cells (CD3^−^ CD19^−^ CD49b^+^ NKp46^+^) in the peripheral blood of EO771 tumour-bearing mice treated with vehicle or ruxolitinib (90 mg kg^−1^ BID). Bars show the mean±s.e.m. Vehicle *n*=6, ruxolitinib *n*=7. FACS analyses were performed on tissue samples collected from mice treated as described in Fig. 2a (protocol II) for the EO771 model and as in Fig. 3a for the 4T1.2 model. (**e**) Quantification of lung metastasis in *Rag2*^*−/−*^*γc*^*−/−*^ mice that were intravenous (i.v.)-injected with 2x10^5^ EO771 cells. Mice were subjected, or not (control), to adoptive transfer of NK cells. The number of metastases in the big lobe from individual mice, and the mean±s.e.m. are shown. Control *n*=4, with NK cells *n*=5 mice.

To characterize JAKi effects on the immune system, we analysed the immune profile of tumour-bearing mice treated with ruxolitinib. Spleens from ruxolitinib-treated EO771 and 4T1.2 mice were significantly smaller than controls (Fig. 4a). The numbers of B and T cells were decreased in both models and the myeloid population was decreased in the 4T1.2 model (Supplementary Fig. 4A). A major effect of JAKi treatment was a reduction in the NK-cell population (Fig. 4b). The effect on NK cells was systemic since they were also reduced in bone marrow and in peripheral blood of JAKi-treated tumour-bearing mice (Fig. 4c,d). To prove the importance of NK cells in controlling metastatic growth, we performed adoptive transfer of NK cells in *Rag2*^*−/−*^*γc*^*−/−*^ *mice*, which lack anti-tumour immune responses. Infusion of activated NK cells significantly decreased lung metastasis (Fig. 4e), indicating that they are essential to control metastatic spread of breast cancer cells.

**Figure 5:**
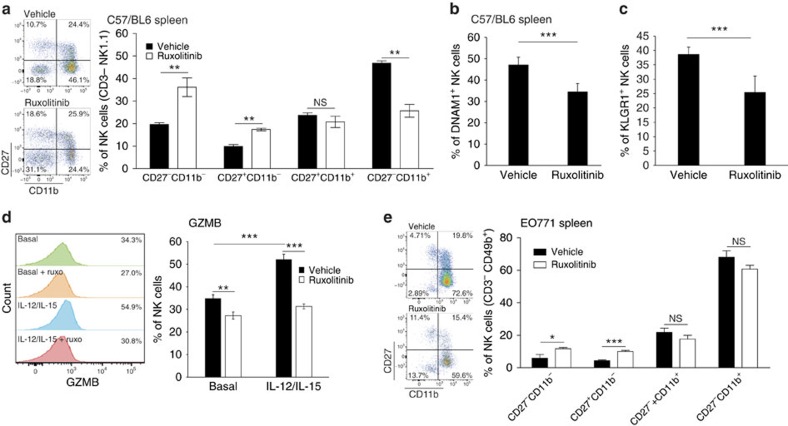
JAKi decrease NK-cell maturation and activation. (**a**) Splenic NK-cell maturation analysis from tumour-free mice after treatment with vehicle or ruxolitinib (90 mg kg^−1^ BID) for 6 days. CD3^−^ NK1.1^+^ NK cells were analysed for CD27 and CD11b expression. Bars show the mean±s.e.m. Representative images of FACS gating are shown. Vehicle *n*=5, ruxolitinib *n*=5. **P<0.01. NS, not significant with two-tailed Mann–Whitney test. (**b**,**c**) Splenic NK cells (CD3^−^ NK1.1^+^) from C57BL/6 mice treated for 6 days with ruxolitinib (90 mg kg^−1^ BID) or vehicle, were stained for DNAM1 and KLRG1 receptors. Bars show the mean±s.d. For DNAM1: vehicle *n*=5, ruxolitinib *n*=4; for KLGR1: vehicle *n*=5, ruxolitinib *n*=5. **P<0.01, ***P<0.005, with two tailed *t*-test. (**d**) MACS-purified and IL-2 expanded NK cells were pretreated 3 h with vehicle or 0.5 μM ruxolitinib and stimulated for 1 h with IL-12 (5 ng ml^−1^) and IL-15 (50 ng ml^−1^). Granzyme B (GZMB) expression was detected by FACS and representative images are shown. Data are expressed as percentage of positive NK cells, and the mean±s.d. of a technical triplicate is shown. **P<0.01, ***P<0.005 with two tailed *t*-test. (**e**) Splenic NK-cell maturation analysis from EO771 tumour-bearing mice treated with vehicle or ruxolitinib (90 mg kg^−1^ BID) as described in Fig. 2a (protocol II). CD3^−^ CD49b^+^ NK cells were analysed for CD27 and CD11b expression and representative images of FACS gating are shown. Bars show the mean±s.e.m. Vehicle *n*=7, ruxolitinib *n*=8. *P<0.05, ***P<0.005 with two tailed Mann–Whitney test.

NK-cell maturation and activation are dependent on JAK/STAT signalling^28^,^37^. In line with this, ruxolitinib treatment of non-tumour-bearing mice skewed the pattern of NK-cell maturation defined by CD11b and CD27 expression^38^,^39^ (Fig. 5a), and reduced the percentage of NK cells expressing the maturation markers DNAM1 and KLGR1 (Fig. 5b,c). Moreover, in cultures of primary mouse NK cells, treatment with ruxolitinib prevented cytokine-induced Granzyme B and Perforin expression (Fig. 5d; Supplementary Fig. 4B), indicating a defect in NK-cell activation following JAK inhibition. Notably, ruxolitinib treatment caused the same effect in tumour-bearing mice, since it enhanced the proportion of NK cells with an immature phenotype (Fig. 5e).

**Figure 6:**
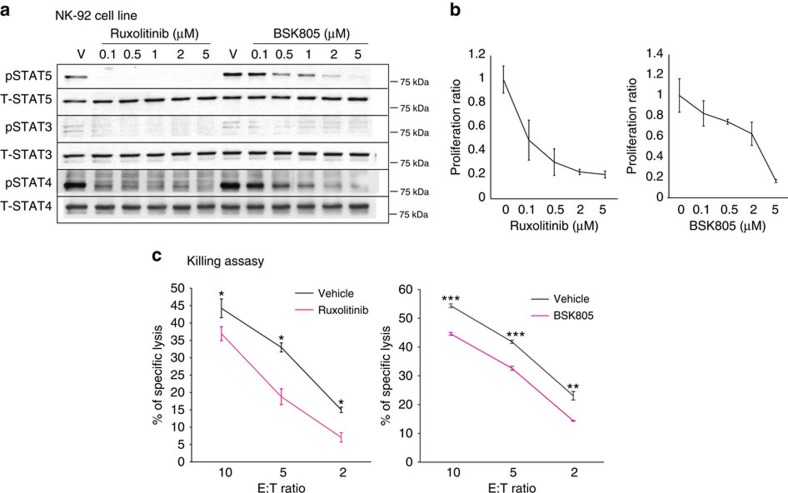
Inhibition of the JAK/STAT pathway blocks proliferation and cytotoxicity of NK cells. (**a**) Western blot analysis of pSTAT5, pSTAT3 and pSTAT4 levels in the human NK cell line, NK-92, after 6 h of treatment with different concentrations of ruxolitinib or BSK805. Total (T) STAT5, STAT3 and STAT4 levels are also shown. (**b**) Proliferation of NK-92 cells after treatment with different concentrations of ruxolitinib for 4 days or BSK805 for 3 days. Data, expressed as mean±s.d. of technical replicates (ruxolitinib *n*=5, BSK805 *n*=4), are given as the ratio of proliferating cells compared with vehicle. (**c**) Cytotoxicity of NK-92 cells against HeLa cells, after 6 h of culture, was analysed by flow cytometry. NK-92 cells were pretreated for 3 h with 0.5 μM ruxolitinib, or 0.5 μM BSK805 or vehicle, then counted and mixed with HeLa cells at the indicated effector–target ratio (E:T). Data are expressed as percentage of NK-92 killing ability; the mean±s.d. of technical replicates is shown. *P<0.05, **P<0.01, ***P<0.005 with two tailed *t*-test.

To investigate the direct impact of JAKi on human NK cells, we employed the NK-92 cell line, which shows STAT3, STAT4 and STAT5 activation (Fig. 6a). Ruxolitinib treatment blocked STAT phosphorylation and strongly decreased proliferation of NK cells (Fig. 6a,b). Interestingly, decreased pSTATs and proliferation were also observed with the specific JAK2i, BSK805 (ref. 40; Fig. 6a,b). In cytotoxicity assays, treatment with both JAKi significantly decreased the killing ability of NK cells against carcinoma cells (Fig. 6c), indicating that JAK/STAT pathway inhibition impairs the anti-tumour potential of NK cells.

### JAK2 activation is essential for NK-cell tumour surveillance

**Figure 7:**
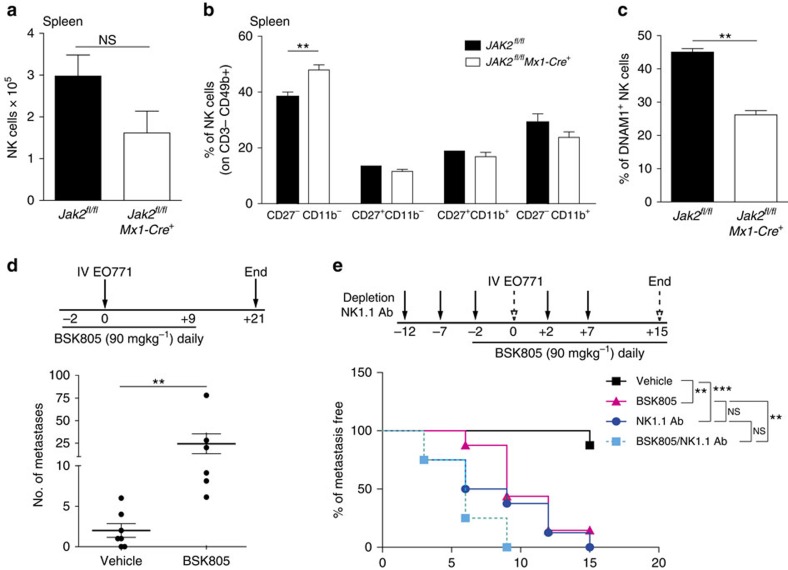
JAK2 activation is essential for NK-cell-mediated anti-tumour immunity. (**a**) Quantification of NK cells (CD3^−^ CD49b^+^) in spleens from *JAK2*^*fl/*fl^ and *JAK2*^*fl*/fl^ *Mx1-Cre* mice. Bars show the mean±s.e.m. *JAK2*^*fl/fl*^ *n*=6, *JAK2*^*fl/fl*^ *Mx1-Cre n*=5. (**b**) NK-cell maturation stages in spleens from *JAK2*^*fl/fl*^ and *JAK2*^*fl/fl*^ *Mx1-Cre* mice; CD3^−^ CD49b^+^ NK cells were analysed for CD27 and CD11b expression. Bars show the mean±s.e.m. of the splenic NK-cell percentage for each gate. *JAK2*^*fl/*fl^ *n*=6, *JAK2*^*fl/fl*^ *Mx1-Cre n*=5. (**c**) Quantification of DNAM1^+^ NK cells (CD3^−^ CD49b^+^) in spleens from *JAK2*^*fl/*fl^ and *JAK2*^*fl/fl*^ *Mx1-Cre* mice. Bars show the percentage±s.e.m. *JAK2*^*fl/fl*^ *n*=6, *JAK2*^*fl/fl*^ *Mx1-Cre n*=5. (**d**) Top: schematic representation of the treatment schedule. Bottom: quantification of lung metastasis after i.v. injection of 2x10^5^ EO771 tumour cells and treatment with vehicle or BSK805 (90 mg kg^−1^ daily) from day −2 to day +9. The number of metastases in the whole lung from individual mice and the mean±s.e.m are shown. Vehicle *n*=7, BSK805 *n*=6. (**e**) Top: schematic representation of the treatment schedule. Bottom: Kaplan–Meier curves show the incidence of metastasis detected by bioluminescent imaging performed every 3 days. Mice were treated with NK1.1 Ab (100 μg per mouse) at day −12, −7, −2, +2 and +7 from i.v. injection of EO771 tumour cells on day 0. BSK805 (90 mg kg^−1^) was administered daily either alone, or in combination with the NK1.1 antibody, from day −2 until the end of the experiment. *n*=8 mice per group. Log-rank (Mantel-Cox) Test: vehicle versus BSK805 **P=0.002, vehicle versus NK1.1 Ab ***P=0.0003, BSK805 versus NK1.1 Ab NS. *P*=0.2952, BSK805 versus BSK805/NK1.1 Ab **P=0.0085, NK1.1 Ab versus BSK805/NK1.1 Ab NS. *P*=0.2405.

To demonstrate that inhibition of JAK2 is sufficient to decrease NK-cell function, we used the *Mx1-Cre JAK2*^*fl/fl*^ mouse model^41^. We found that splenic NK-cell number, maturation, and expression of the activating receptor DNAM1 were all reduced on JAK2 deletion (Fig. 7a–c). To define the role of JAK2 in the prometastatic effect of JAKi, we used the JAK2i BSK805 in tumour-bearing mice. BSK805 mimicked the JAK2 knockout phenotype, hence causing a reduction in NK-cell number, which was due to decreased proliferation, and an altered maturation profile (Supplementary Fig. 5A–C). Importantly, blocking JAK2 alone was sufficient to increase metastatic burden (Fig. 7d), even though BSK805 decreased pSTAT3 levels in metastases (Supplementary Fig. 5D). These data provide evidence that JAK2 is required for an active NK-cell-mediated anti-tumour response.

To confirm the pivotal role of NK cells in JAK2i-mediated metastasis enhancement, we compared the effect of BSK805 with that of NK-cell depletion on metastasis formation. Anti-NK1.1 Ab treatment, which completely eliminates NK cells (Supplementary Fig. 6A), mimicked the effect of the JAK2i in accelerating lung metastasis incidence (Fig. 7e). Importantly, the concomitant use of BSK805 did not further significantly enhance metastasis incidence, or metastatic burden, compared with depletion alone (Fig. 7e; Supplementary Fig. 6B), meaning that NK-cell inhibition is the main cause of JAK2i-enhanced metastasis.

### IL-15 overcomes the JAK2i-mediated increase of metastasis

**Figure 8:**
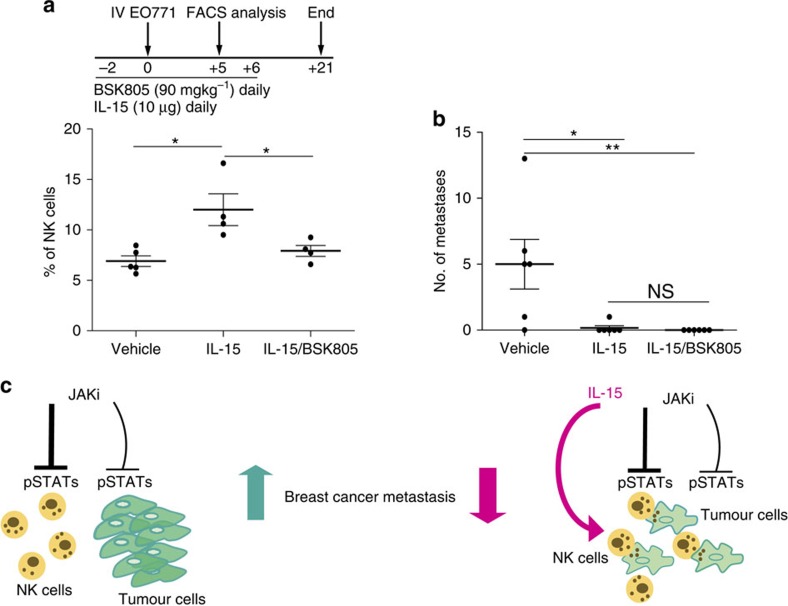
Immunostimulation with IL-15 prevents the JAKi-mediated increase of metastasis. (**a**) Top: schematic representation of the treatment schedule. 4x10^5^ EO771 tumour cells were injected i.v. and mice were treated with IL-15 (10 μg per mouse) or the combination of IL-15 and BSK805 (90 mg kg^−1^ daily) from day −2 to day +6. Bottom: quantification of NK cells (CD3^−^ NK1.1^+^) in the peripheral blood collected by tail vein puncture from randomly chosen mice at day +5 after i.v. injection of tumour cells. Bars show the percentage of circulating NK cells in individual mice and the mean±s.e.m for each group. Vehicle *n*=5, IL-15 *n*=4 and IL5 plus BSK805 *n*=4. *P<0.05 with Mann–Whitney test. (**b**) Quantification of lung metastases at the end of the experiment. The number of metastasis in the whole lung from individual mice and the mean±s.e.m are shown. *n*=6 mice per group. *P<0.01, **P<0.001, NS, not significant with Dunn's Multiple Comparison Test. (**c**) STATs are active in metastatic tumour cells, and in NK cells in the tumour environment. Left: JAK/STAT pathway inhibition in NK cells weakened anti-tumour immunosurveillance by reducing proliferation, maturation and NK-cell activation. The immunomodulatory properties of JAKi prevail over potential anti-tumour effects of blocking STAT activation in cancer cells, thus leading to enhanced metastasis. Right: the combinatorial treatment of a JAKi with the immunostimulator IL-15 blocks metastasis formation, potentially by boosting NK-cell function through multiple JAKs.

IL-15 is a potent immunostimulator that enhances expansion and activation of NK cells^42^,^43^. In searching for approaches to overcome the immunosuppressive effect of JAKi, we investigated whether enhancing NK-cell function through IL-15 stimulation, would reduce metastasis in the context of JAK2 inhibition. To focus our analysis on the initial phase of metastasis dissemination and growth, IL-15 and BSK805 were administered in combination starting on day −2 and continuing until day +6 after intravenous (i.v.) injection of EO771 tumour cells. IL-15-treated mice showed an increased NK-cell population and strongly reduced metastasis (Fig. 8a,b). Importantly, IL-15 addition to BSK805 prevented metastasis, even though BSK805 decreased IL-15-induced NK-cell expansion (Fig. 8a,b). This result indicates that immunostimulation with IL-15 can override the effect of JAK2i on enhancing metastasis (Fig. 8c), despite the reduction of the NK-cell population due to JAK2 inhibition.

## Discussion

The rationale for targeting the JAK/STAT pathway in cancer is provided by the positive outcome of clinical trials employing ruxolitinib for the treatment of myeloproliferative neoplasms^7^,^8^,^9^ and pancreatic cancer^44^. Moreover, in different preclinical settings, JAKi blocked the growth of basal-like breast cancer^5^ and overcame resistance driven by other targeted therapies^6^,^45^. In a *Pten*-deficient prostate cancer model, characterized by massive infiltration of immunosuppressive myeloid cells, JAK2 inhibition decreased tumour growth and restored anti-tumour immunity^46^. Several studies suggest that the anti-inflammatory effects of ruxolitinib contribute to its clinical benefits^44^,^46^,^47^. However, multi-JAKi also have immunosuppressive activity^48^,^49^,^50^, and an increased risk of viral and bacterial infections in treated patients has been observed^51^. In patients with myeloproliferative neoplasms, infections during ruxolitinib treatment were linked to decreased NK-cell frequency, maturation and activation^49^. Since NK cells are important players in controlling tumour progression and metastatic spread^28^,^33^,^52^,^53^,^54^, inhibiting their function might have adverse clinical consequences in solid cancers. The JAK/STAT pathway is active in several lymphocytic and myeloid populations, which are affected by treatment with JAKi^2^,^48^,^50^ (Supplementary Fig. 4A). We show that the inhibitory effect of JAKi on NK cells has a pivotal role in increasing metastasis formation, however, we cannot exclude that additional cell types in the immune compartment contribute to the observed phenotype.

Immune-mediated responses in malignancy are complex and diverse, such that the consequences of interfering with different immune cells at various stages of tumour progression might be context dependent^21^,^55^. Although the employment of JAKi to target tumour-promoting inflammation represents a valuable therapeutic strategy^44^,^46^, protection against cancer is critically dependent on robust immunosurveillance^21^,^28^,^56^. In our work, we demonstrate that JAKi block activation of multiple STATs that are essential for NK-cell-mediated anti-tumour effects^32^,^33^. The inhibition of NK-cell-mediated tumour immunosurveillance overrides potential anti-tumour effects of blocking the activated JAK/STAT pathway in breast cancer cells, thereby enhancing experimental metastasis. These results suggest that JAK pathway activation in the immune compartment might be beneficial in cancer. In support of this, JAK2 expression was associated with a decreased risk of tumour recurrence in a large cohort of breast cancer patients^57^. JAK2 messenger RNA was high in the non-epithelial fraction of tumours and correlated with levels of TILs^57^, suggesting that activation of the JAK pathway in TILs is important to elicit the anti-tumour response.

Targeted therapy, developed to block aberrantly activated pathways in cancer cells, can have unexpected effects by concomitantly blocking the same pathway in cells of the tumour stroma. Indeed, other kinase inhibitors have been shown to have immunosuppressive properties and unintentionally block anti-tumour immune response^58^,^59^. Our study highlights the importance of evaluating the effect of targeted therapy in the tumour environment in addition to the cancer cell compartment to prevent potential harmful bystander effect.

Here we show that treatment with IL-15 overcame the enhancement in metastasis caused by the immunosuppressive effect of JAK2 inhibition, despite its effect on reducing the NK-cell population. It is possible that IL-15, which can signal through JAK1 and JAK3 (refs 60, 61), is able to boost NK-cell activity in the context of JAK2 inhibition by activating multiple JAKs. In conclusion, our study indicates that JAKi enhance metastasis in experimental models of breast cancer by inhibiting NK-cell functions (Fig. 8c), and that JAK2 has an essential role in controlling NK-cell-mediated tumour immunosurveillance. Moreover, we propose that to overcome potential unintentional effects of targeted therapies, interventions counteracting bystander immunosuppression, such as use of immunostimulators (Fig. 8c) or adoptive immunotherapy^62^ might improve clinical benefits of JAKi.

## Methods

### Patient material

The study and its design were approved by the institutional review board (Ethikkommission beider Basel, decision 93/12). According to the local laws at the time of biopsy (before 2014), the patients were asked to give a general informed consent to conduct studies on their material, provided all diagnostic analyses had been completed and the material would not be exhausted for study purposes; the study strictly adhered to both prerequisites. We analysed 34 tissue samples, from 14 patients, consisting of paired primary breast cancer biopsies and subsequent bone metastases biopsies, collected between 2004 and 2011 at the Institute of Pathology of the University Hospital, Basel. All cases were reviewed and classified according to the WHO 2012 criteria. Tissue samples were formalin-fixed and paraffin-embedded. The immunohistochemical staining was performed as described^63^ with the following antibodies: phosphotyrosine (p) STAT3 (Tyr705; Cell Signaling, 9145) and pSTAT5 (Tyr694; Cell Signaling, 9359). OEstrogen receptor (ER), progesterone receptor (PR) and HER2 status were determined with antibodies routinely used for diagnostics (ER: Ventana/Roche, 790-4324; PR, Ventana/Roche, 790-4296; and HER2 Ventana/Roche, 790-2991). The immunoreactive score was calculated by multiplying the percentage of positive cell-nuclei (PP) by the staining intensity (SI). The PP score was assigned as 0: <1%, 1: 1–20%, 2: 21–50%, 3: 51–80%, 4: >80% positive nuclei. The SI score was assigned as 0: negative, 1: weak, 2: moderate, 3: strong. The TIL infiltration score was assigned as 0: negative for TILs, 1: single cell detected in a biopsy, 2: maximum of 5% of cells in a biopsy are TILs. In the case of duplicate biopsies from the same patient, the average of PP and SI was calculated before assigning the immunoreactive score. To quantify TIL infiltration, the average of the TIL score for pSTAT3 and pSTAT5 for each biopsy was used. In duplicate biopsies for the same patient, the average TIL score was calculated to assign the final score. The investigator was blinded for the immunohistochemistry quantifications.

### Animal experiments

Mice were housed under hygienic conditions according to the Swiss guidelines governing animal experimentation and experiments were approved by the Swiss veterinary authorities (Kantonales Veterinäramt Basel-Stadt, Switzerland, license 2419). All the mice used in the study were 6–8 weeks old females. *Jak2*^*f/f*^ *Mx1-Cre* mice^41^ were bred and maintained at the University of Veterinary Medicine, Vienna under pathogen-free conditions according to Federation of Laboratory Animal Science Associations guidelines, and experiments were conducted according to Austrian law (licenses BMWFW-68.205/0093-WF/V/3b/2015 and BMWF-68.205/0218-II/3b/2012). *Jak2*^*f/f*^ *Mx1-Cr*e mice were generated by crossing *Mx1-Cre*^*+*^ mice^64^ and *Jak2*^*f/f*^ mice^65^ and treated i.p. with 200 μg poly(I:C) (InvivoGen) every 3 days for 2 weeks to induce gene deletion.

For intracardiac injection, 1.3 × 10^5^ 1833 cells were injected in athymic nude mice and 5 × 10^5^ EO771 cells were injected in C57BL/6 mice in 100 μl PBS. Cells were collected from subconfluent cultures, washed in PBS and injected into the left ventricle using a 26G needle syringe. We confirmed successful injection of tumour cells by the intracardiac route: (1) by detecting arterial blood pumping into the syringe when properly inserted in the heart; (2) by *in vivo* bioluminescent imaging of injected tumour cells detectable throughout the mouse. For i.v. injection 2 × 10^5^ or 4 × 10^5^ EO771 cells were injected into the tail vein. Metastatic burden was monitored and quantified by *in vivo* bioluminescence over the course of the experiment. Bioluminescent imagining and quantification were performed with the NightOwl (Berthold technologies) or the IVIS (PerkinElmer). Alternatively, lungs were placed into Bouin's solution and the number of nodules was counted.

For NK-cell depletion, the NK1.1 (PK136) antibody^66^ was administered at 100 μg per mouse in 100 μl PBS by intraperitonial (i.p.) injection every 5 days. Recombinant human IL-15 (Immunex, Seattle, WA) was administered, at 10 μg per mouse, in 100 μl PBS by i.p. injection for 6 days.

For orthotopic injections, 5 × 10^6^ 1833 or 1x10^6^ EO771 cells (in 100 μl of PBS) were injected into the fourth mammary fat pad. Treatment with ruxolitinib (90 mg kg^−1^ BID) was started once tumours were palpable and continued for 2–3 weeks. 4T1.2 cells (1 × 10^5^ in 100 μl of PBS) were injected in the fourth mammary fat pad of BALB/C or NSG mice. After 2 days, mice were randomized into two groups receiving vehicle or ruxolitinib (90 mg kg^−1^ BID). Treatments were continued until the primary tumour reached 1,000 mm^3^ in volume (∼3 weeks) or signs of distress were observed. Tumour volumes were determined according to the formula: length × (diameter)^2^ × *π*/6. At the end of the experiment, lungs, bones and blood were collected to quantify metastases and circulating tumour cells. Lungs were placed into Bouin's solution and nodule number was counted, or H&E staining was performed on paraffin-embedded lung sections and the metastatic area was quantified. The metastatic index was calculated as the number of lung foci/tumour volume, or as area of metastasis/lung total area. Metastases in the bone were quantified by FACS analysis as EPCAM^+^ cells, or by colony-forming assays, done by grinding long bones in a mortar and plating cell suspensions in 6-well culture dishes in α-MEM. The 4T1.2 were selected with 60 μM 6*-*thioguanine for 1 week^67^, and then single colonies were counted after fixation and crystal violet staining.

Blood burden assays were performed to quantify circulating tumour cells. Blood was collected from the right atrium by heart puncture with a 25-gauge needle syringe containing 0.1 ml of heparin. Blood was plated in tissue culture medium and 2 days later tumour cells were selected with 60 μM 6-thioguanine for 1 week, and then colonies were counted. Circulating tumour cells were calculated as the total number of colonies in the dish divided by the volume of blood taken.

For the adoptive transfer experiment, 2 × 10^6^ Magnetic-activated cell sorting (MACS)-purified and IL-2 expanded NK cells were injected in *Rag2*^*−/−*^*γc*^*−/−*^ in C57BL/6 mice, purchased from Taconic Biosciences. Two days later, 2 × 10^5^ EO771 cells were inoculated by i.v. injection. After 15 days lungs were collected and placed into Bouin's solution for nodule counting.

### Histology of mouse tissues

Primary tumours, long bones and lungs were collected 2–3 h after the last treatment and fixed in 10% neutral buffered formalin for 24 h at 4 °C. Long bones were decalcified in 0.5 M pH7.5 EDTA, for at least 5 days at 4 °C, before paraffin embedding. Sections of 7 μm in thickness for bone, and 5 μm in thickness for primary tumours and lungs were cut and immunohistochemistry for pSTAT3 (1:50, Cell Signaling, 9145) and pSTAT5 (1:50, Cell Signaling, 9359) was performed with the Ventana Discovery XT biomarker platform (Roche Diagnostics). Quantification of the PP tumour cells was performed by counting the number of positive- and total cell-nuclei at × 40 magnification. Different fields were quantified for each sample and the sum was used to calculate the PP tumour cells for each mouse. The investigator was not blinded for the immunohistochemistry quantifications.

### Cell culture and proliferation assays

The MDA-MB-231 scp1833 (1833) cell line was provided by J. Massagué (Memorial Sloan Kettering Cancer Center, New York) and has been authenticated by STR profiling. HeLa cells were obtained from ATCC. 1833 and HeLa cells were cultured in Dulbecco's modified Eagle's medium (DMEM) supplemented with 10% fetal bovine serum (SIGMA) at 37 °C, 5% CO_2_. The EO771 cell line was obtained, with the approval of F.M. Sirotnak (Memorial Sloan Kettering Cancer Center, New York), from A. Schrum (Mayo Clinic, Rochester, Minnesota); cells were cultured in Iscove's Modified Dulbecco's Medium (IMEM) supplemented with 10% fetal bovine serum (SIGMA) at 37 °C, 5% CO_2_. The 4T1.2 cell line was provided by R. Anderson (Peter MacCallum Cancer Centre, Melbourne) and cultured in αMEM (Life technology, 12561) supplemented with 10% fetal bovine serum (SIGMA) at 37 °C, 5% CO_2_. The NK-92 cell line was provided by M. Stern (Department of Biomedicine, University Hospital Basel) and cultured in αMEM supplemented with 12.5% horse serum (GIBCO Invitrogen), 12.5% fetal bovine serum (SIGMA), 0.2 mM inositol, 0.1 mM 2-mercaptoethanol, 0.02 mM folic acid and 100 U ml^−1^ rhIL-2 (Novoprotein, C013) at 37 °C, 5% CO_2_. Splenic mouse NK cells were isolated using DX5-labelled MACS beads according to the manufacturer's instructions (Miltenyi) and cultured in RPMI (SIGMA) supplemented with 10% fetal calf serum, 0.1 mM 2-mercaptoethanol and 5000 U ml^−1^ rhIL-2 (Proleukin, Novartis) for 5days at 37 °C, 5% CO_2_. All cell lines were regularly tested for mycoplasma contamination with the LookOut Mycoplasma PCR Detection Kit (Sigma).

For *in vivo* studies, the 1833 and EO771 cell lines were transduced with a lentiviral vector encoding Luc-2eGFP genes (L2G) as described^68^ for the expression of luciferase and GFP proteins.

For proliferation assays, 1833 and EO771 (1.5 × 10^3^) cells or NK-92 (4 × 10^4^) cells were seeded in quadruplicates in 96-well plates, in complete medium. After 24 h, cells were treated for 72 or 96 h with increasing concentrations of NVP-BSK805 (BSK805) or ruxolitinib in DMEM supplemented with 5% serum. Cell proliferation was evaluated by crystal violet staining and absorbance measurement at 595 nm.

### Western blot analysis

Tumour cell lines were treated for 10 min with IL-6 (50 ng ml^−1^) and different concentration of JAK inhibitors, as indicated in the figure legends, after starvation in 0.5% serum. NK-92 cells were maintained in normal medium and treated for 6 h with different concentrations of JAK inhibitors. Protein lysates were made with NP40 lysis buffer and processed as described^69^. For western blot analysis, the following antibodies were used: pSTAT3 (1:1,000, Cell Signaling, 9145), pSTAT4 (1:500 BD, 612738), pSTAT5 (1:1,000 Cell Signaling, 9359), STAT3 (1:1,000 BD, 610190) STAT4 (1:1,000, Cell Signaling, 2653), and STAT5 (1:500)^70^. The original scan of the western blots are shown in Supplementary Fig. 7.

### Kinase inhibitors

NVP-BSK805 (BSK805) and ruxolitinib (INCB018424) were provided by Novartis. For *in vitro* studies, 10 mM stock solutions were prepared in DMSO. For oral administration, BSK805 was freshly formulated in 5% NMP, 80% PEG300, 15% Solutol HS, protected from light and administered to mice at 5 ml kg^−1^. Ruxolitinib was formulated in 0.5% hydroxypropyl-methyl-cellulose and administered to mice at 10 ml kg^−1^.

### FACS analysis

Organs were collected from mice and single-cell suspensions were made from the spleen, bone marrow and peripheral blood. Total viable cell numbers were determined using an Automated Cell Viability Analyzer (Vi-CELL, Beckman Coulter). The number of cells in immune subpopulations in bone and spleen was calculated by normalizing their percentage to the total organ cell number. Samples were analysed with FACSAria (Becton Dickinson) after staining with the following antibodies from BioLegend or eBioscience: CD3 (17A2), CD19 (*6D5*), NKp46 (29A1.4), NK1.1 (PK136), EpCAM (G8.8), CD45 (30-F11), CD27 (LG.3A10), CD11b (M1/70) CD49b (DX5), DNAM1 (10E5), KLRG1 (2F1), Granzyme B (GB11) and Ki-67 (SolA15). Intracellular staining for Granzyme B and Ki-67 were performed with the Foxp3/Transcription Factor Staining Buffer Set (eBioscience), following the manufacturer's instructions.

### Real-time PCR

NK-cell stimulations were performed in the presence of 5,000 U ml^−1^ rhIL-2, 5 ng ml^−1^ recombinant murine IL-12 (rmIL-12) (R&D) and 50 ng ml^−1^ rmIL-15 (PeproTech). RNA was extracted with the Qiagen RNAeasy microkit (Qiagen) and complementary DNA prepared with the iScript synthesis kit (BioRad). Real-time PCR was performed with the following primers: *GrzmB*-fw 5′-CCAATCAGATATGTGCGGG-3′, *GrzmB*-rev 5′-GGAAACTATGCCTGCAGCC-3′, *Prf*-fw 5′-GATGTGAACCCTAGGCCAGA-3′ *Prf*-rev 5′-GGTTTTTGTACCAGGCGAAA-3′ and the housekeeping gene *Rplp0*-fw 5′-CCTGGCATTGTCTGTGGAGAC-3′, *Rplp0*-rev 5′-GCTTCAGCTTTGGCAGGG-3′.

### *In vitro* cytotoxicity assay

A FACS-based cytotoxicity assay was performed by mixing HeLa and NK-92 cells for 4–6 h. NK-92 cells were pretreated with ruxolitinib (0.5 μM) or BSK805 (0.5 μM). After treatment, NK-92 cells were counted and mixed at different effector–target (E:T) cell ratios with HeLa cells labelled with Cell Proliferation Dye eFluo 670 (eBioscience). After 4–6 h of incubation at 37 °C, cell death was quantified by FSC-A SSC-A living/dead gate. The % of specific lysis was measured as [% eFluo 670^*+*^ dead cells after co-incubation with NK cells]−[% eFluo 670^+^ dead cells without addition of NK cells].

### Statistical methods

All the *in vivo* experiments were performed in biological replicates, as reported in the figure legends. The number of mice was calculated by performing small pilot experiments and the mice in the experiments were not randomized. Group allocation and outcome assessment were not performed in a blinded manner. No exclusion/inclusion criteria were applied for the analysis of mice experiment; if mice were euthanized during the experiment due to tumour burden they were included until measurements were available. For *in vitro* experiments, the statistics were performed on technical replicates and representative experiments are shown. Statistical analyses were performed with GraphPad PRISM software. Depending on the type of experiment, data were tested using two-tailed Student's *t*-test, tailed Mann–Whitney test, or with the Dunn's multiple comparison test as reported in the figure legends. Differences were considered significant if the *P* value was ≤0.05 (*P≤0.05, **P<0.01, ***P<0.005). Data from patient biopsies were analysed with the Spearman's test and with the exact Wilcoxon's rank-sum test from R package (https://www.r-project.org/).

### Data availability

All the relevant data that support the findings of this study are available from the corresponding author on request.

## Additional information

**How to cite this article**: Bottos, A. *et al.* Decreased NK-cell tumour immunosurveillance consequent to JAK inhibition enhances metastasis in breast cancer models. *Nat. Commun.* 7:12258 doi: 10.1038/ncomms12258 (2016).

## Supplementary Material

Supplementary Information Supplementary Figures 1-7 and Supplementary Table 1

Peer Review
