# Supplementary material for: Decreased NK-cell tumour immunosurveillance consequent to JAK inhibition enhances metastasis in breast cancer models
Source: Nat Commun. 2016 Jul 13;7:12258. doi: 10.1038/ncomms12258 (PMC4947169; doi:10.1038/ncomms12258)
Supplement: Supplementary Information — Supplementary Figures 1-7 and Supplementary Table 1 [file ncomms12258-s1.pdf]

Supplementary Figures

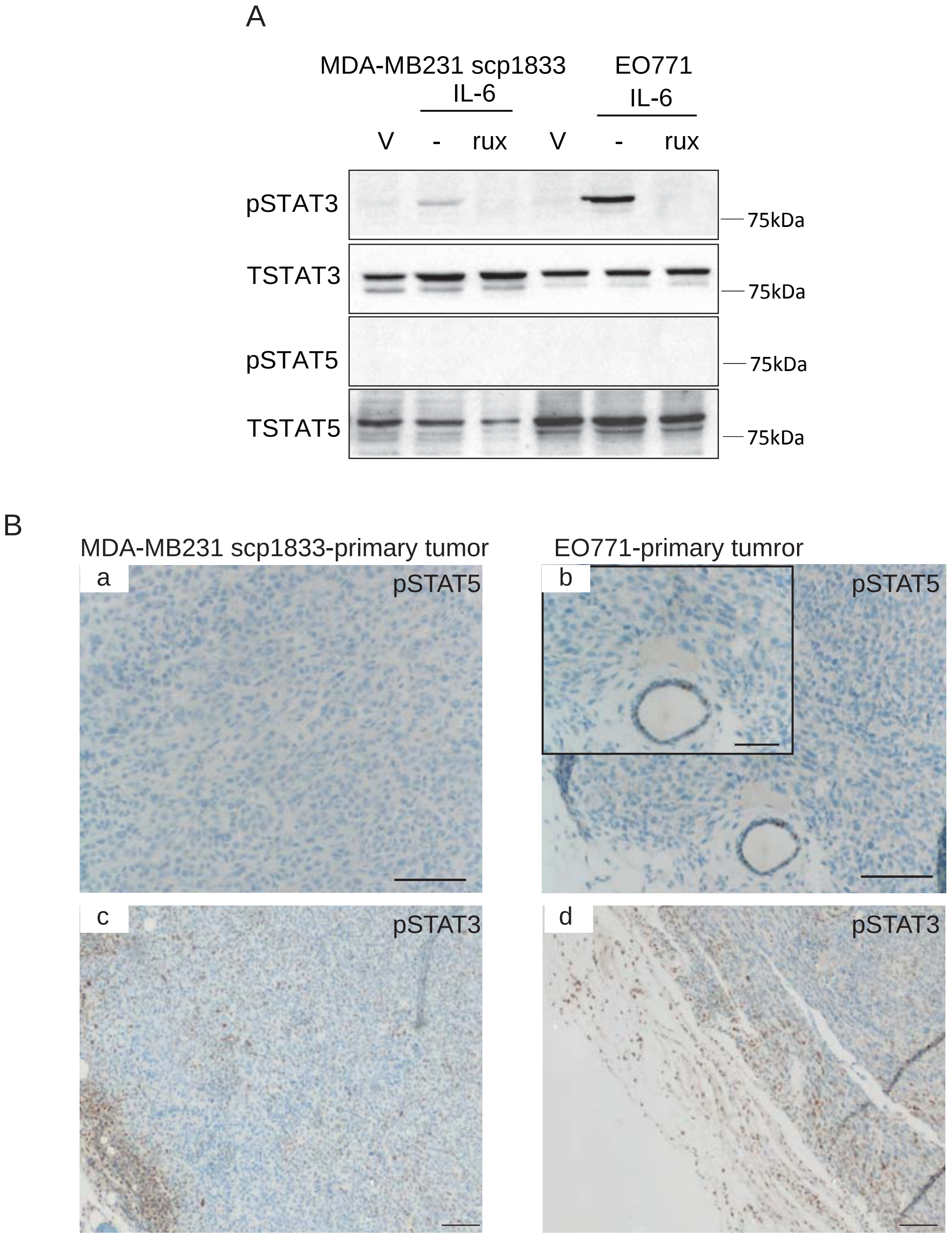

Supplementary Figure 1: Activation of the JAK/STAT pathway in breast cancer models.
A) Western blot analysis for pSTAT3 and pSTAT5 in lysates of 1833 and EO771 cancer cells after treatment, or not, with IL-6 (50ngml^{-1}), ruxolitinib (2µM) or vehicle. The blots were also probed for total (T) STAT3 and STAT5 levels. **B)** Histological analysis of 1833 and EO771 mammary tumors. a-b: Representative images of pSTAT5 staining. As a positive control, pSTAT5 epithelial cells in the normal mammary gland are shown in the higher magnification. c-d: Representative images of pSTAT3 staining. Both sections show the external ring of the primary tumor where pSTAT3 positive TILs are detected. Scale bar 100µm, for higher magnification 50µm.

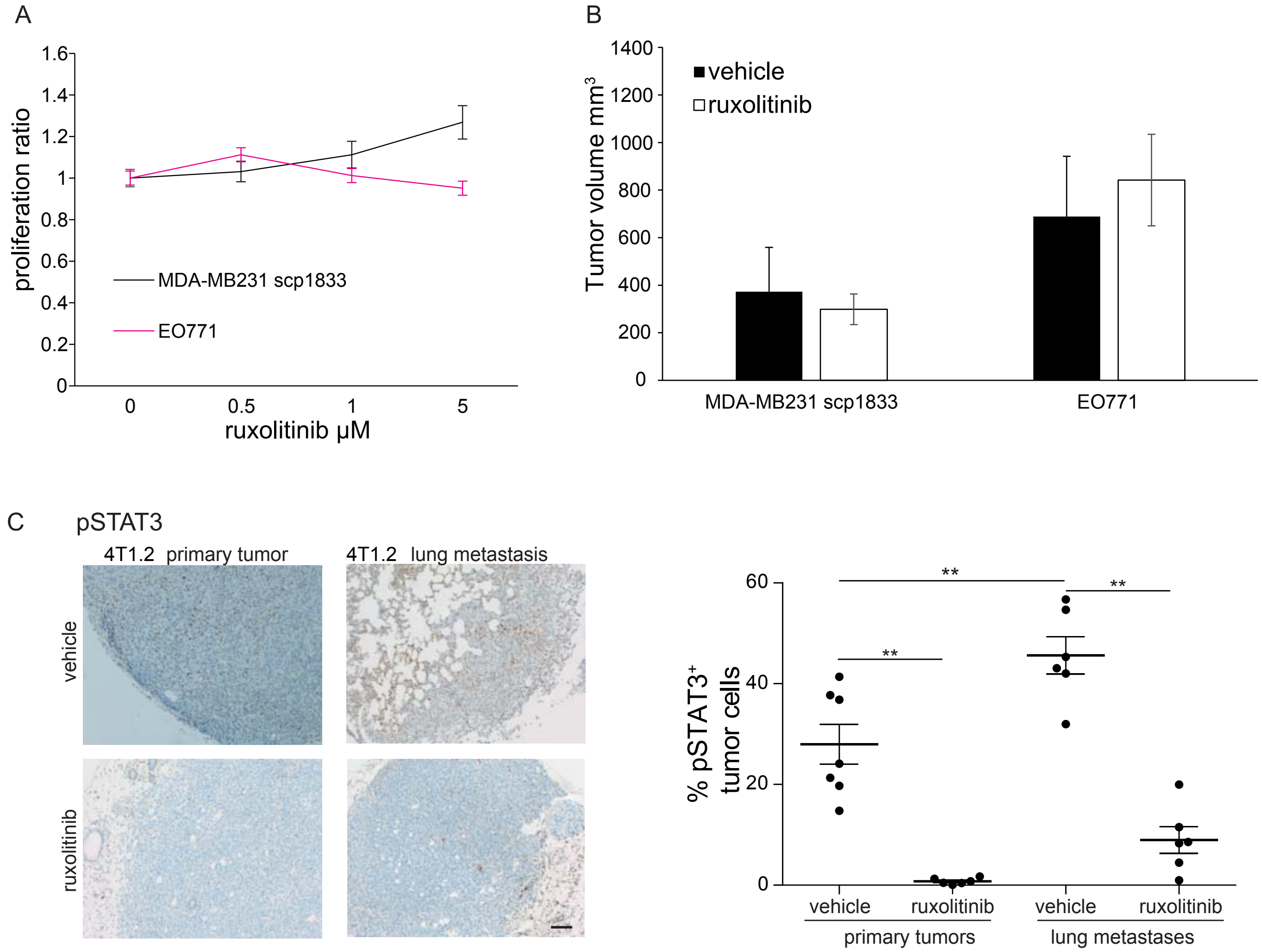

Supplementary Figure 2: Effect of ruxolitinib on cell proliferation and primary tumor growth.
A) In vitro proliferation of 1833 and EO771 cell lines treated 4 days with different concentrations of ruxolitinib. Data are expressed as ratio of proliferating cells compared to vehicle; the mean +/- SD of technical triplicates is shown. **B)** Quantification of primary mammary tumor volume (mm^3) after treatment with ruxolitinib ($90 mgkg^{-1}$ BID) for 3 weeks (1833) or 10 days (EO771). Bars show the mean +/- SEM. For 1833 n=6 per group; for EO771 vehicle n=4, ruxolitinib n=6. **C)** Left: Representative images of pSTAT3 staining in a 4T1.2 primary mammary tumor and lung metastasis from vehicle- or ruxolitinib-treated mice. Scale bar 100µm. Right: Quantification of percentage of positive pSTAT3 tumor cells in 4T1.2 primary tumors and lung metastases from mice treated with vehicle or ruxolitinib ($90 mgkg^{-1}$ BID). The percentage of positive pSTAT3 tumor cells from individual mice and the mean +/- SEM are shown. For primary tumors: vehicle n=7, ruxolitinib n=6; for lung metastases: vehicle n=6, ruxolitinib n=6. ** $p<0.01$, with two tailed Mann Whitney test.

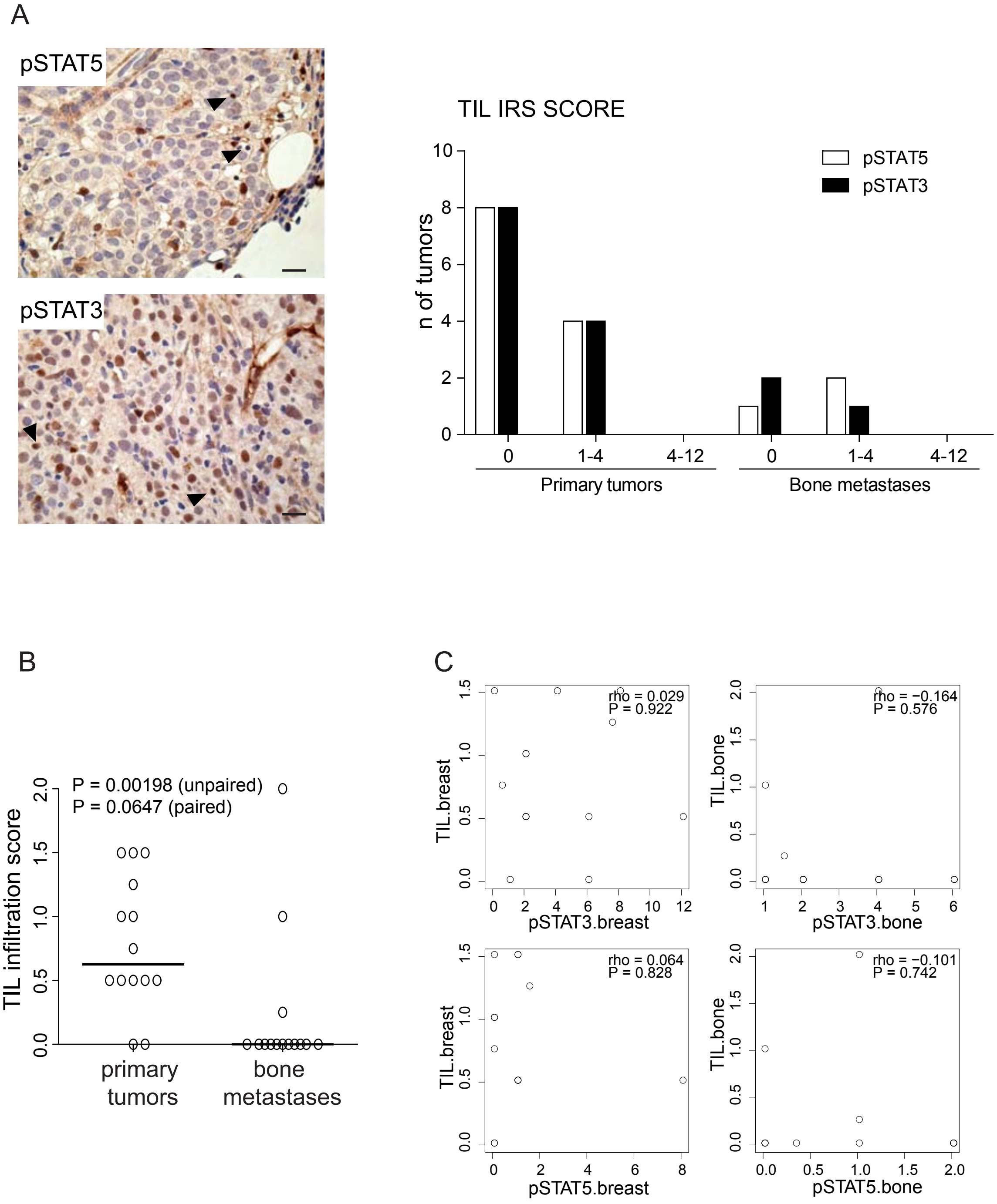

Supplementary Figure 3: Activation of STATs in TILs in breast cancer patients.

A) Left: Representative images of pSTAT5 and pSTAT3 staining in the primary tumors of breast cancer patients. Black arrow heads indicate TILs. Scale bar 20µm. Right: Histogram of pSTAT3 and pSTAT5 immunoreactive score (IRS) in TILs from primary tumors and paired bone marrow metastases positive for TIL infiltration. Tumors were scored according to their pSTAT3 and pSTAT5 IRS in TILs in the IRS range (0, 1-4, and 4-12). **B)** TIL infiltration score for individual primary tumors and bone metastases and the median are shown. The TIL infiltration score in the primary tumors and bone metastases were compared as matched (paired test) and as unmatched (unpaired test). The results show a reduction of TIL infiltration in the bone metastases, compared to the primary tumors (unpaired test). The reduction of TIL infiltration in metastases is independent of TIL infiltration in the matched primaries (paired test). The analysis was performed with the exact Wilcoxon rank sum test; the P values are shown. **C)** Correlation analysis between levels of pSTAT5 and pSTAT3 staining in cancer cells and TIL infiltration in primary tumors and bone metastases. The analysis was performed with the Spearman test; P values and Spearman's rank correlation coefficients (rho) are shown. n=14 patients.

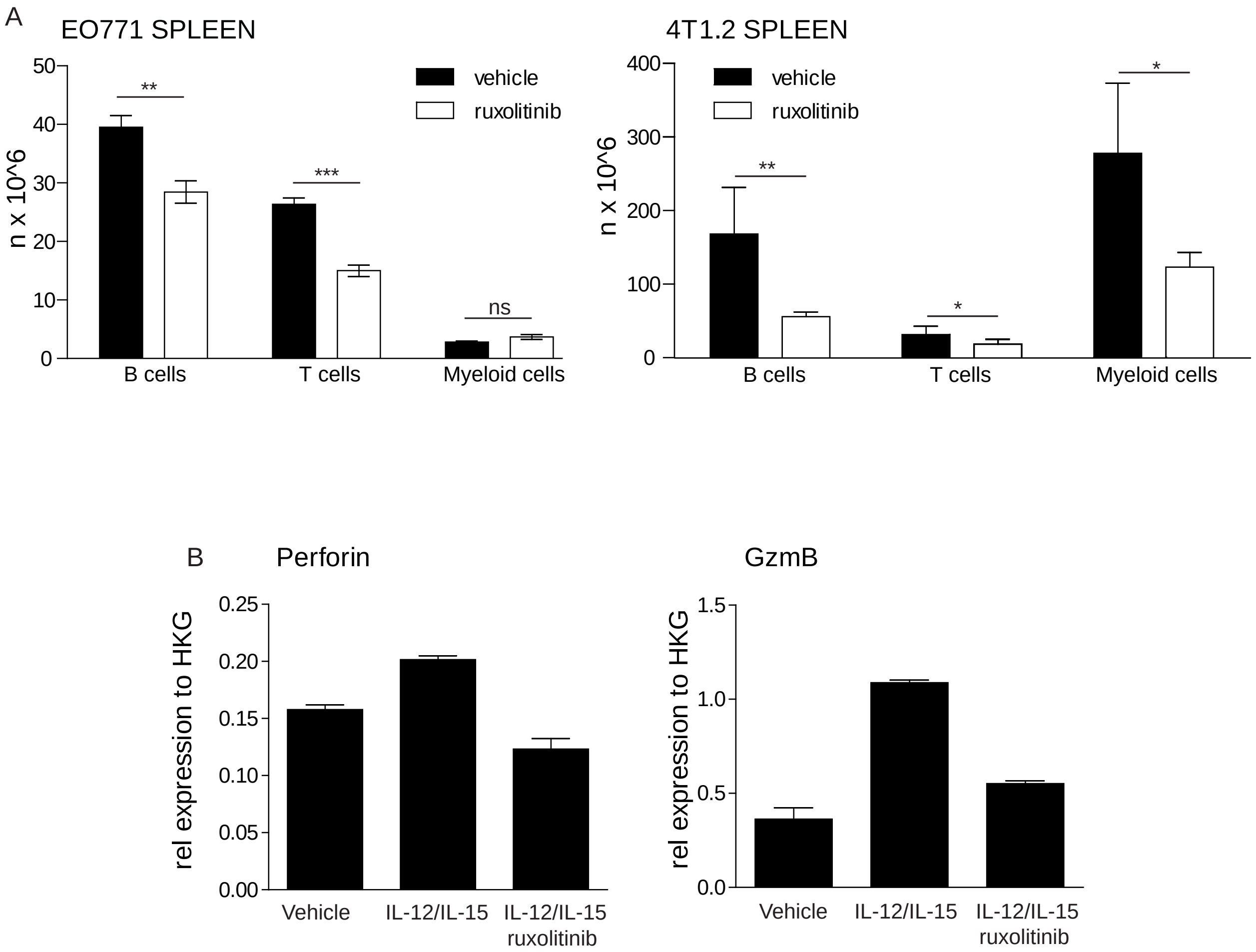

Supplementary Figure 4: Effect of JAKi on immune cells.

A) Quantification of B cells ($CD19^+$), T cells ($CD3^+$) and myeloid cells ($CD11b^+$) by FACS analysis in spleens from EO771 and 4T1.2 tumor-bearing mice treated with vehicle or ruxolitinib (90mgkg^{-1} BID) as in Fig. 2A (protocol II) and 3A, respectively. Bars show the mean +/- SEM. For EO771 vehicle n=7, ruxolitinib n=8; for 4T1.2: vehicle=7, ruxolitinib=6. *p<0.05, ** p<0.01, with two tailed Mann Whitney test. **B)** MACS-purified and IL-2 expanded NK cells were stimulated with IL-12 (5ngml^{-1}) and IL-15 (50ngml^{-1}) for 1 hour after pretreatment with vehicle or 0.5µM ruxolitinib for 3 hours. Prf1 and Gzmb mRNA levels were determined by qPCR and quantified as relative expression to the house keeping gene (HKG). The mean +/- SEM of a technical duplicate is shown**.**

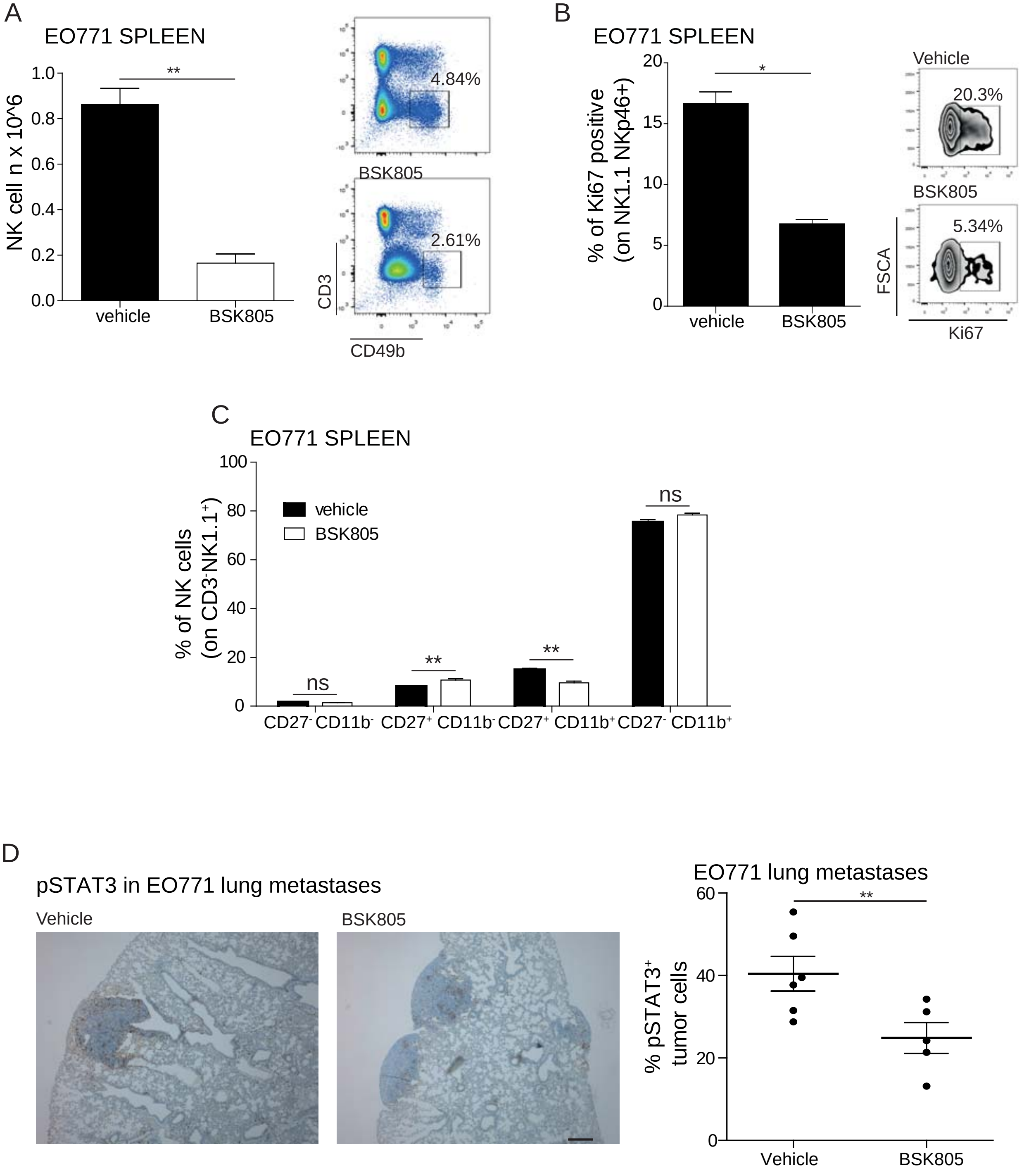

Supplementary Figure 5: JAK2i decreases NK cells and enhances metastasis.

A) Quantification of NK cells ($CD3^-$ $NK1.1^+$) in spleens of EO771 tumor-bearing mice after treatment with vehicle or BSK805 ($90mgkg^{-1}$) for 11 days. Bars show the mean +/- SEM. Vehicle n=5, BSK805 n=6. Representative images of NK cell-gating for the FACS analysis are shown. **B)** Quantification of Ki67 positive NK cells (gated on $NK1.1^+$ $NKp46^+$) in spleens from EO771 tumor-bearing mice treated with vehicle or BSK805 ($90mgkg^{-1}$) for 13 days. Representative images of FACS gating are shown. Bars show the mean +/- SEM. Vehicle n=5, BSK805 n=5. **C)** NK cell maturation stages in spleens from EO771 tumor-bearing mice after treatment with vehicle or BSK805 ($90mgkg^{-1}$) for 11 days. $CD3^-CD49b^+$ NK cells were analyzed for CD27 and CD11b expression. Bars show the mean +/- SEM. Vehicle n=5, BSK805 n=6. **D)** Left: Representative images of pSTAT3 in EO771 lung metastasis from vehicle- or BSK805-treated mice. Scale bar 100µm. Right: Quantification of the percentage of positive pSTAT3 tumor cells in lung metastases from EO771 IV injected mice treated with vehicle or BSK805 ($90mgkg^{-1}$). The percentage of positive pSTAT3 tumor cells from individual mice and the mean +/- SEM are shown. Vehicle n=6, BSK805 n=5. For all the experiments * $p<0.05$, ** $p<0.01$, ns=not significant with two tailed Mann Whitney test.

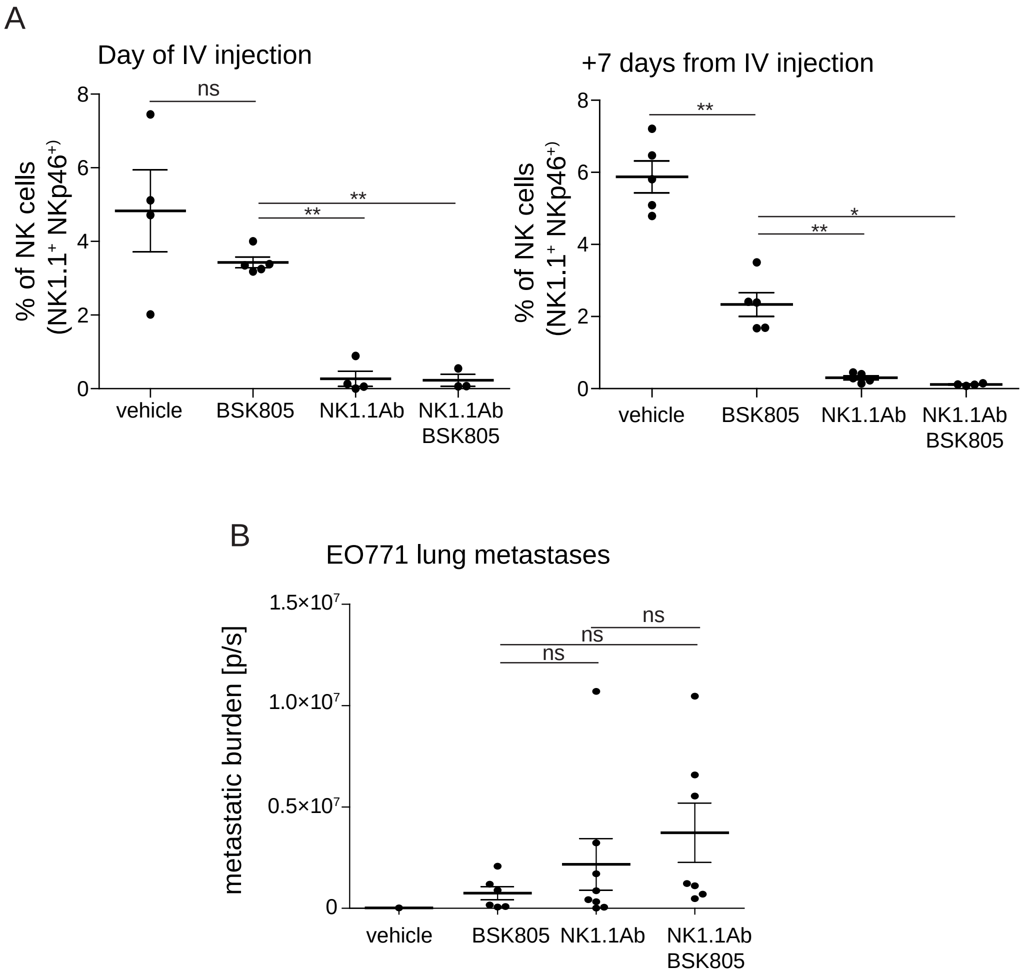

Supplementary Figure 6: NK cells have a pivotal role in JAK2i-mediated metastasis enhancement.

A) Left: Quantification of NK cells ($CD3^-$ $NK1.1^+$ $NKp46^+$) in peripheral blood collected by tail vein puncture from randomly chosen mice the day of EO771 IV injection (day 0). Vehicle n=4, BSK805 n=5, NK1.1 Ab n=4, NK1.1 Ab plus BSK805 n=3. Right: Quantification of NK cells ($CD3^-$ $NK1.1^+$ $NKp46^+$) in the peripheral blood isolated by tail vein puncture from randomly chosen mice 1 week after EO771 IV injection (day +7). Mice were treated with BSK805 (90mgkg^{-1}) and/or Anti-NK1.1 Ab (100µg) as in Fig. 7E. Vehicle n=6, BSK805 n=5, NK1.1 Ab n=5, NK1.1 Ab plus BSK805 n=6.The percentage of peripheral NK cells for individual mice and the mean +/- SEM are shown. **B)** Quantification of lung metastases in mice IV injected with 2x10^5 EO771 cells and treated with BSK805 (90mgkg^{-1}) and/or Anti-NK1.1 Ab (100µg) as in Fig. 7E. The bioluminescence (Total Flux [p/s]) of lungs from individual mice positive for metastasis at day 15 and the mean +/- SEM is shown. Vehicle n=1, BSK805 n=6, NK1.1 Ab n=8, NK1.1 Ab plus BSK805 n=7.For all the experiments * p<0.05, ** p<0.01, ns=not significant with two tailed Mann Whitney test.

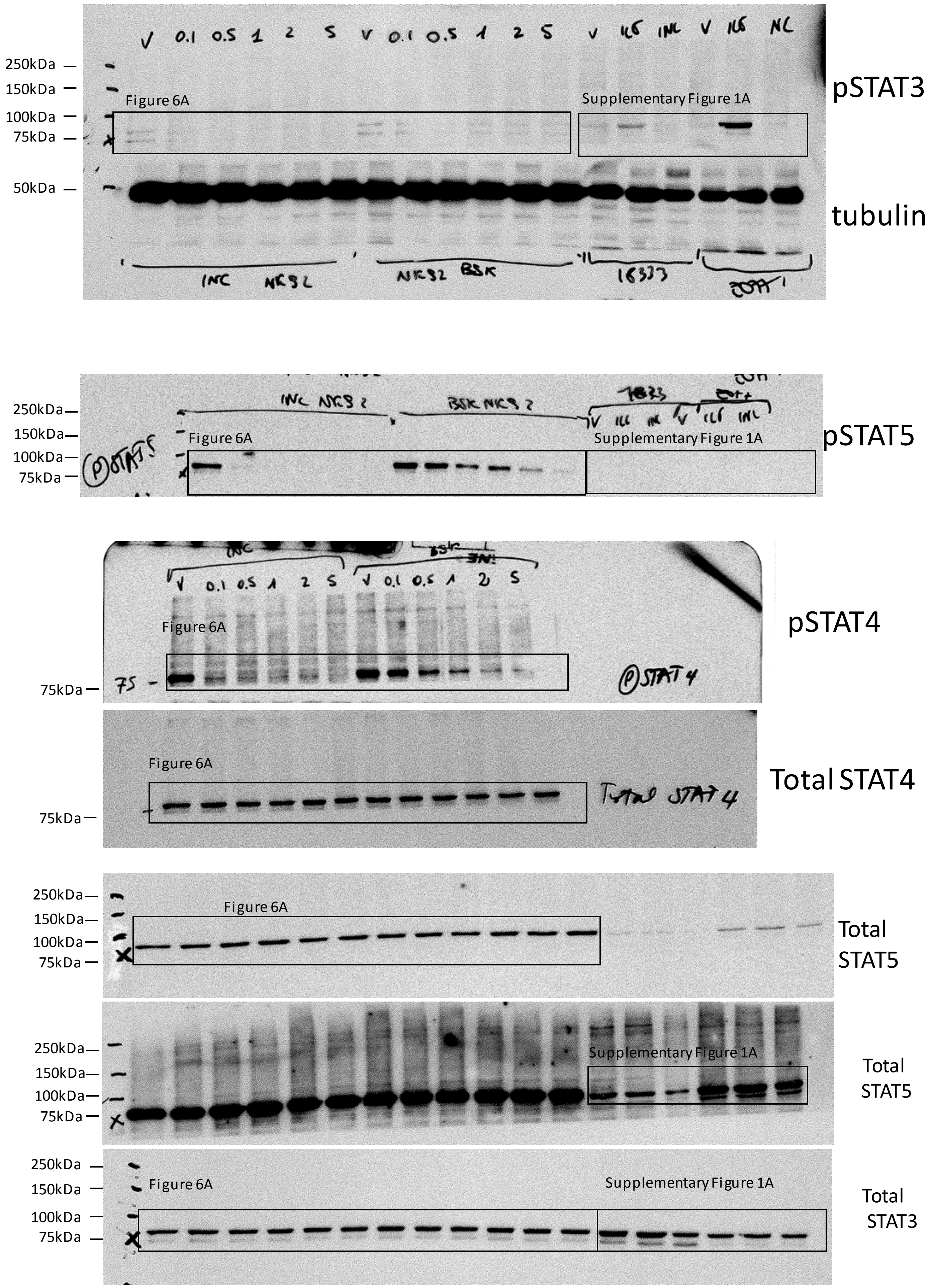

Supplementary Figure 7: Full scans of western blot.
The original full western blot scan are shown. The black boxes indicate the cropped displayed area in the corresponding figure.

Patient number		Pathology	ER	PR	HER2	pSTAT3 tumor cells PP	pSTAT3 tumor cells SI	pSTAT3 TILs infiltration	pSTAT3 TILs PP	pSTAT3 TILs SI	pSTAT5 tumor cells PP	pSTAT5 tumor cells SI	pSTAT5 TILs infiltration	pSTAT5 TILs PP	pSTAT5 TILs SI
1	Breast	Ductal	pos	pos	neg	0	0	0	0	0	0	0	0	0	0
	Breast		pos	focal pos	NA	30	1	1	15	2	0	0	2	5	2
	Bone		pos	neg	NA	50	3	0	0	0	0	0	0	0	0
2	Breast	Ductal G1	pos	pos	neg	30	1	0	0	0	100	2	1	0	0
	Bone		pos	pos	neg	50	2	0	0	0	NA	NA	NA		
3	Breast	Lobular G2	pos	NA	NA	15	2	0	0	0	5	1	1	0	0
	Bone		pos	neg	neg	25	2	2	5	3	2	1	2	5	2
4	Breast	Lobular G3	pos	pos	neg	100	2	1	0	0	1-2	1	2	2	2
	Bone		pos	neg	NA	5	1	0	0	0	0	0	0	0	0
	Bone		pos	<5%	NA	20	2	0	0	0	5	2	1	30	3
5	Breast	Ductal G2	pos	neg	neg	100	3	1	0	0	15	1	0	0	0
	Bone		pos	weak	NA	30	2	0	0	0	10	2	0	0	0
6	Breast	Lobular G2	pos	pos	neg	40	2	1	10	1	5-10	1	2	10	2
	Bone		pos	pos	NA	60	2	0	0	0	15	1	0	0	0
7	Breast	Ductal G3	pos	neg	neg	10	2	1	0	0	0	0	1	0	0
	Bone		neg	neg	neg	10	1	0	0	0	10	2	0	0	0
8	Breast	Ductal G2	pos	<5%	NA	10	2	1	0	0	0	0	1	0	0
	Bone		pos	neg	neg	10	2	0	0	0	0	0	0	0	0
9	Breast	Ductal G2	pos	pos	NA	15	2	0	0	0	1-2	1	1	0	0
	Bone		pos	neg	neg	20	1	0	0	0	20-50	1	0	0	0
10	Breast	Ductal G2	pos	pos	neg	0	0	2	1	3	0	0	1	0	0
	Bone		pos	pos	neg	10 focal	2	0	0	0	0	0	0	0	0
11	Breast	Mucinous G1	pos	pos	neg	15	1	0	0	0	0	0	0	0	0
	Bone		pos	<1%	NA	2	1	1	0	0	0	0	1	0	0
12	Breast	NA	pos	pos	neg	35	2	0	0	0	0	0	0	0	0
	Breast			neg	NA	90	2	0	0	0	0	0	0	0	0
	Bone		pos	pos	neg	10	1	0	0	0	1-25	2	0	0	0
13	Breast	Lobular G2	pos	pos	neg	70	2	1	0	0	0.001	1	1	0	0
	Breast				NA	80	3	1	10	3	10	2	2	5	2
	Bone		pos	pos	amplified	50	2	0	0	0	5	1	0	0	0
	Bone				NA	10	2	0	0	0	0	0	0	0	0
	Bone				NA	50	2	0	0	0	0	0	0	0	0
14	Breast	Lobular G2	pos	pos	neg	60	2	0	0	0	5	1	1	5	2
	Bone		pos	weak	NA	15	2	0	0	0	1	2	0	0	0

Supplementary Table 1: Description of breast cancer patients' paired biopsies.
The characteristics of primary tumors and matched bone metastases for 14 breast cancer patients are listed. For some patients, more than one biopsy was analyzed. ER, PR and HER2 status were assigned by immunohistochemistry. The percentage of positive (PP) tumor cells and the staining intensity (SI) for pSTAT3 and pSTAT5 for each biopsy are shown. The TIL infiltration score, the PP TIL and the SI for pSTAT3 and pSTAT5 in TILs for each biopsy are shown. NA=Not available.
