# Supplementary material for: Decreased NK-cell tumour immunosurveillance consequent to JAK inhibition enhances metastasis in breast cancer models
Source: Nat Commun. 2016 Jul 13;7:12258. doi: 10.1038/ncomms12258 (PMC4947169; doi:10.1038/ncomms12258)
Supplement: Peer Review [file ncomms12258-s2.pdf]

Editorial Note: this manuscript has been previously reviewed at another journal that is not operating a transparent peer review scheme. This document only contains reviewer comments and rebuttal letters for versions considered at Nature Communications.
